# Putative Familial Transmissible Bacteria of Various Body Niches Link with Home Environment and Children’s Immune Health

**DOI:** 10.1128/Spectrum.00872-21

**Published:** 2021-12-08

**Authors:** Lu Zhao, Wanning Chen, Yongsheng Ge, Xin Lv, Ying Wang, Han Yu, Yi Liu, Dingfeng Wu, Na Jiao, Yuanqi Wu, Daqing Lv, Guoqing Zhang, Fuzhong Xue, Xiaohong Xu, Zhongtao Gai, Ruixin Zhu, Lei Zhang, Guoping Zhao

**Affiliations:** a Shandong Children’s Microbiome Center, Pediatric Research Institute, Qilu Children’s Hospital, Cheeloo College of Medicine, Shandong University, Jinan, People’s Republic of China; b Department of Biostatistics, School of Public Health, Cheeloo College of Medicine, Shandong University, Jinan, People’s Republic of China; c National Institute of Health Data Science of China & Institute for Medical Dataology, Cheeloo College of Medicine, Shandong University, Jinan, People’s Republic of China; d Department of Gastroenterology, The Shanghai Tenth People’s Hospital, Department of Bioinformatics, School of Life Sciences and Technology, Tongji University, Shanghai, People’s Republic of China; e Bioinformatics Division, GloriousMed Clinical Laboratory Co., Ltd., Shanghai, People’s Republic of China; f Laboratory Medicine, Qilu Children's Hospital, Cheeloo College of Medicine, Shandong University, Jinan, People’s Republic of China; g Guangdong Institute of Gastroenterology, Guangdong Provincial Key Laboratory of Colorectal and Pelvic Floor Diseases, Department of Colorectal Surgery, the Sixth Affiliated Hospital, Sun Yat-sen University, Guangzhou, People’s Republic of China; h Research Institute, GloriousMed Clinical Laboratory Co., Ltd., Shanghai, People’s Republic of China; i CAS Key Laboratory of Computational Biology, Bio-Med Big Data Center, Shanghai Institute of Nutrition and Health, University of Chinese Academy of Sciences, Chinese Academy of Sciences, Shanghai, People’s Republic of China; j Shanghai Shenyou Biotechnology Co. Ltd., Shanghai, People’s Republic of China; k CAS Key Laboratory of Synthetic Biology, Shanghai Institutes for Biological Sciences, University of Chinese Academy of Sciences, Chinese Academy of Sciences, Shanghai, People’s Republic of China; l Hangzhou Institute for Advanced Study, University of Chinese Academy of Sciences, Hangzhou, People’s Republic of China; m Central Laboratory, the First Affiliated Hospital of Weifang Medical University, Weifang, People’s Republic of China; Lerner Research Institute

**Keywords:** transmission, microbiome, family generation, gut, mouth, skin

## Abstract

Owing to their significant impact on children’s long-term health, familial factors in the microbiomes of children have attracted increasing attention. However, the mechanism underlying microbiome transmission across generations remains unclear. A significantly lower alpha diversity was observed in the gut flora of children than in the gut flora of parents and grandparents; the alpha diversity of oral and skin microbiota was relatively higher in children than in their predecessors. Gut, oral, and skin microbiome was more similar between family members than between unrelated individuals. Meanwhile, 55.05%, 61.09%, and 76.73% of amplicon sequence variants (ASVs) in children’s gut, oral, and skin microbiomes, respectively, were transmitted from all family members. Among these, the most transmissible ASVs belonged to *Methylophilaceae*, *Solimonadaceae*, *Neisseriaceae*, and *Burkholderiaceae*, which were defined as “putative familial transmissible bacteria.” Furthermore, we found that the time spent with parents/grandparents and children’s dietary preferences were important factors that influenced the proportion of the transmissible microbiome. Moreover, the majority of transmissible ASVs (85.06%), especially those of *Ruminococcaceae* and *Lachnospiraceae*, were significantly associated with the immune indices, such as CD3^+^, CD4^+^, CD8^+^, IgG, and IgA.

**IMPORTANCE** Our study revealed that the children’s microbiota was partially transmitted from their family members and specific putative transmissible ASVs were associated with the immune system of children. These findings suggest that home life plays a key role in the shaping of young children’s microbiomes and has long-term health benefits.

## INTRODUCTION

A myriad of microorganisms, collected into microbiomes, broadly colonize diverse niches in the human body, including the gut, skin, and mouth. Studies have reported that microbiome homeostasis plays a critical role in maintaining health and that dysbiosis is linked to many diseases, such as periodontal disease (1), metabolic disorders (2, 3), immunopathy (4), and colorectal cancer (5). Furthermore, the microbiome is an important factor in the induction and development of the immune system during early life (6, 7). Experiments in germfree animal models have shown that gut microbial colonization induces the maturation of gut-associated lymphoid tissue (GALT) (8). The development of the gut microbiome in children has lasting effects on the host, and disorders affecting this process may also affect overall health (9, 10), such as increasing the risk of developing childhood obesity and nonalcoholic fatty liver disease (11).

The microbiome is dynamic, changing rapidly during infancy and early childhood, and can be shaped by the delivery mode, diet, and exposure to various environmental conditions (7). Mother-infant microbiome transmission, which is important in the establishment and development of the infant microbiome, has been widely investigated. Studies have shown that the microbiome can be transmitted to infants through both vaginal delivery and breastfeeding (12–14). However, microbiome transmission patterns between family members have been less explored. Recently, the emerging viewpoint that the microbiota can be transmitted along social networks has attracted increasing attention (15). Some studies have suggested that the transmission of commensal microbes often occurs within the social networks of wildlife, particularly through parenthood (16, 17). Similarly, microbial transmission in humans may also be mediated by physical social contact (e.g., touching), which generally occurs in daily life, especially within households. This emphasizes the importance of family members in the shaping of children microbiome, especially if they live together.

Considering that two- or even three-generation households are often the norm in China (18), the microbiomes of children—which are relatively unstable, especially in their early years—could be shaped by or transmitted from the microbiomes of family members other than mothers through daily interactions and lifestyle habits. From this perspective, members of older generations play indispensable roles in influencing the microbiomes of children by serving as potential origins. Evidence suggests that stochastic changes, rather than founder effects, have a greater influence on gut microbial community assembly over time (19). Thus, studying the microbial transmission from different family members to children could provide insights into the microbiome development in early life and potential effects on children’s health. At present, this important topic remains underexplored.

In this study, we investigated the characteristics of gut, oral, and skin microbiota in three generations of individuals belonging to 24 Chinese families with common ethnicities and similar lifestyles. Based on the foundation of household-based and child-centered, we propose the concept of “putative familial transmissible bacteria,” which is defined as “microbial community transfer from family members to children in the same household.” Associations between putative familial transmissible bacteria and lifestyle factors, such as diet and family time, were also explored.

## RESULTS

### Participants’ information.

We enrolled 144 individuals from 24 families (34 children, 48 parents, and 62 paternal and/or maternal grandparents). Among these, 10 families have two children, which means these children have siblings. The average age of the children, at the time of sample collection, was 4.6 ± 2.7 (range 0 to 12) years. The characteristics of the participants, including basic statistical data regarding the immune indices and the time spent with parents or grandparents, are shown in Table 1. A total of 366 microbial samples were collected from the gut (*n* = 108), mouth (*n* = 114), and skin (*n* = 144). The pedigree charts of these participants, and the sample distribution, are shown in Figure S1.

**TABLE 1:** Characteristics of participants belonging to three generations^*a*^

Characteristic	Child	Mother	Father	PGM	PGF	MGM	MGF
No.	34	24	24	18	19	15	10
Age (y)^*b*^	4.6 ± 2.7	34.3 ± 3.3	35.2 ± 3.3	62.4 ± 4.3	63.4 ± 5.3	59.7 ± 5.3	59.5 ± 5.4
Gender (F/M)	20/14	24/0	0/24	18/0	0/19	15/0	0/10
FT (h/d)		9.7 ± 5.4	8.2 ± 5.3	12.1 ± 7.8	9.6 ± 8.8	6.3 ± 6.3	4.9 ± 5.3
Immunity index							
IgA	1.1 ± 0.6	2.4 ± 0.8	2.4 ± 0.9	2.4 ± 0.6	2.3 ± 0.7	3.0 ± 1.0	2.7 ± 1.0
IgG	8.8 ± 2.1	11.7 ± 2.9	11.7 ± 1.5	10.9 ± 1.8	12.7 ± 1.7	12.7 ± 2.3	12.0 ± 2.1
IgM	1.1 ± 0.5	1.2 ± 0.4	1.0 ± 0.4	1.0 ± 0.4	0.9 ± 0.4	1.0 ± 0.5	0.6 ± 0.3
CD3^+^	70.1 ± 8.0	73.3 ± 8.5	73.8 ± 7.7	73.6 ± 7.8	64.2 ± 12.9	66.6 ± 11.1	67.9 ± 9.0
CD4^+^	36.8 ± 7.5	29.1 ± 7.7	39.2 ± 6.5	43.9 ± 7.6	39.5 ± 10.2	40.3 ± 9.0	33.7 ± 10.5
CD8^+^	26.8 ± 5.6	26.9 ± 8.5	29.8 ± 6.1	26.9 ± 8.7	26.5 ± 9.5	22.6 ± 6.2	30.2 ± 14.3
CD3^−^/CD19^+^	13.9 ± 6.3	8.3 ± 3.1	8.8 ± 3.7	10.0 ± 5.2	9.9 ± 6.6	11.1 ± 4.4	6.9 ± 3.2
CD3^−^/CD56^+^	6.7 ± 4.3	10.5 ± 6.9	11.1 ± 6.2	10.2 ± 5.3	14.7 ± 11.7	13.0 ± 7.2	18.4 ± 7.9

a FT, family time; PGM, paternal grandmother; PGF, paternal grandfather; MGM, maternal grandmother; MGF, maternal grandfather.

b Data are the mean ± standard deviation (SD).

### Characteristics of gut, oral, and skin microbiota across three generations.

**FIG 1:**
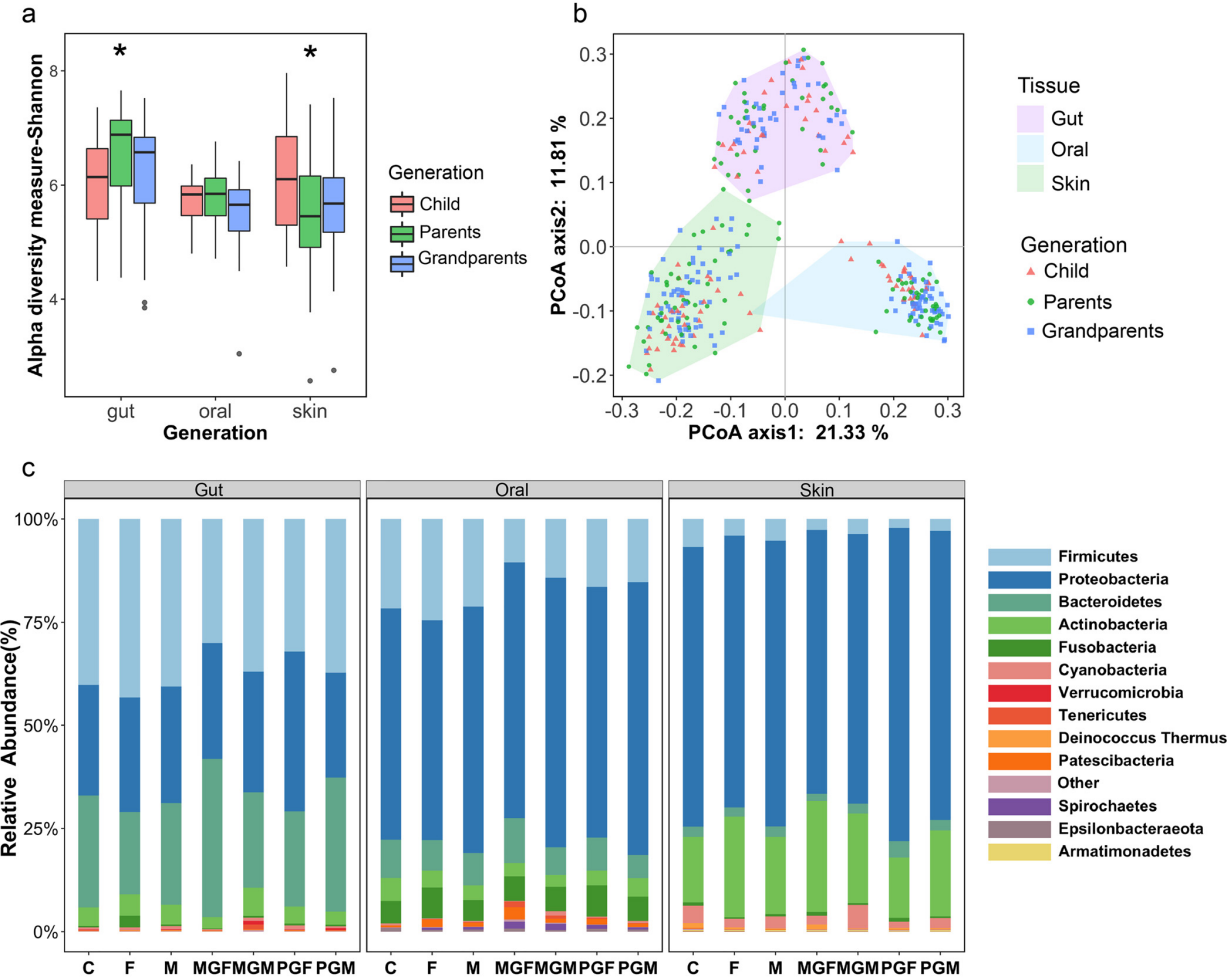
Comparison of bacterial species within three generations. (a) Alpha diversity (Shannon index) of gut, oral, and skin microbiomes for three generations. Analysis of variance (ANOVA) indicated that of the inhabitants of the three microbiomes, the skin microbiota showed the greatest variation between generations (Wilcoxon test, *, *P* < 0.05). (b) Beta diversity based on unweighted UniFrac distances between gut, oral, and skin microbiomes over the course of three generations. (c) Compositions of gut, oral, and skin microbiomes at the phylum level in each household member, summarized with average abundance (C, child; F, father; M, mother; PGM, paternal grandmother; PGF, paternal grandfather; MGM, maternal grandmother; MGF, maternal grandfather).

First, we explored microbiome characteristics under different conditions. When we observed the microbiome distributions among the three generations, we observed a species diversity (Fig. 1a) in the gut flora of children significantly lower than that in the gut flora of their parents and grandparents. In contrast, the alpha diversity of the skin microbiota in children was higher than that in their parents and grandparents. Meanwhile, no significant differences in oral microbiota richness were observed between members of the three generations.

Moreover, differences in microbiota characteristics were observed between ecological niches, where samples were closely clustered (Fig. 1b). Compared to skin and gut microbiota, oral flora showed less variation between populations (Shannon [oral] = 5.65 ± 0.56, Shannon [gut] = 6.25 ± 0.91, Shannon [skin] = 5.72 ± 0.95). At the phylum level (Fig. 1c), the intestinal flora was dominated by *Firmicutes*, *Bacteroidetes*, and *Proteobacteria* in fairly equal proportions. The oral flora was comprised of approximately 50% *Proteobacteria*, while with relatively low percentages of *Firmicutes* and *Bacteroidetes*. However, the skin flora was dominated by *Proteobacteria* and *Actinobacteria*. Despite these differences, the flora structure of the three generations displayed less variation within the same body sites (Fig. 1b and c).

### Family members share similar microbiome profiles.

**FIG 2:**
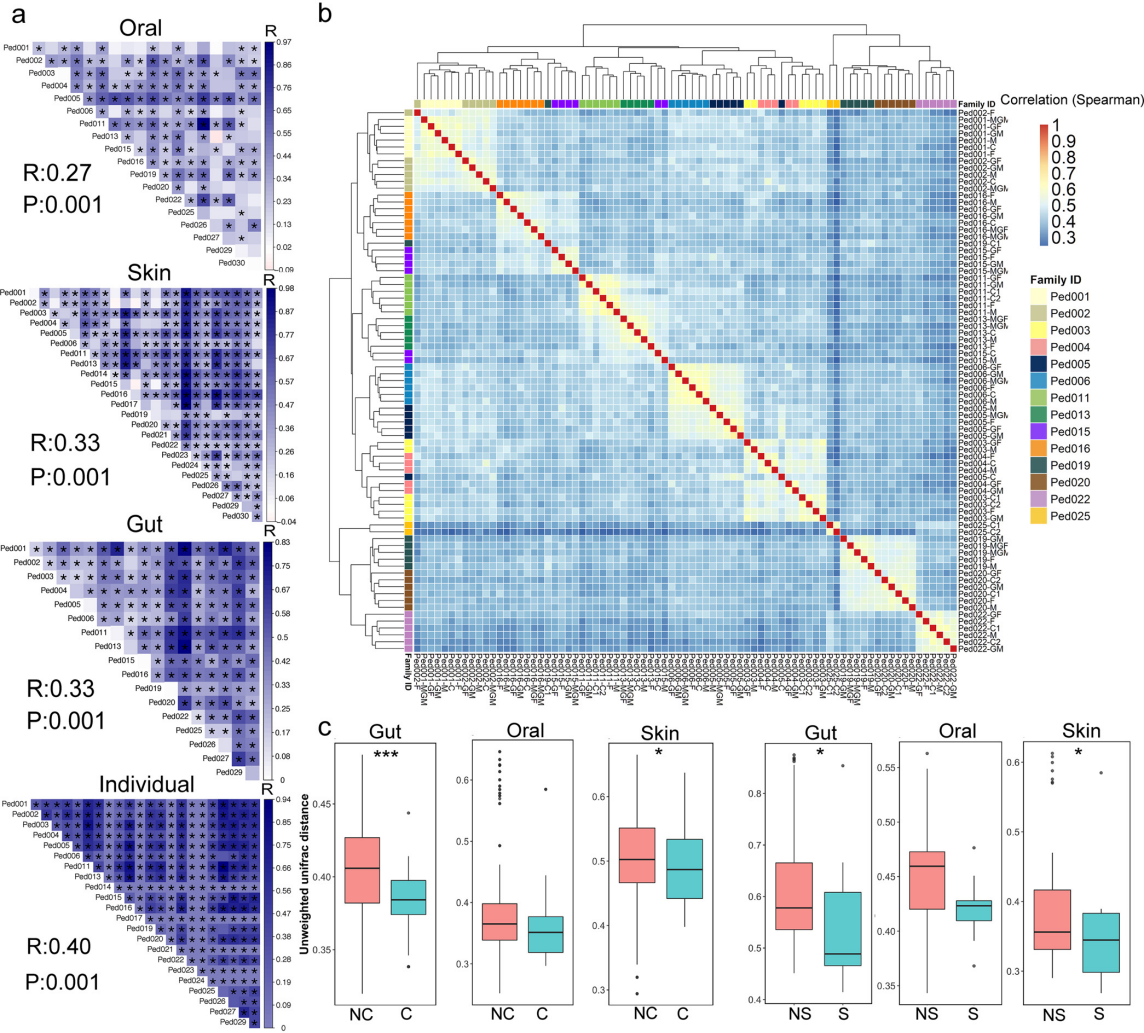
Microbiomes are more similar between family members than between unrelated individuals. (a) Global comparisons (marked “R” and “P” in the lower left corner) and pairwise comparisons (*, *P* < 0.05, heatmap: R) of the flora structures of each ecological niche in each household were performed using ANOSIM, based on the Bray Curtis distance. Larger R values indicate greater variation between households than within households. “Individual” refers to the collection of ASVs gathered from the three ecological niches of each subject. (b) A cluster heatmap of all samples, based on familial differential ASVs. The Kruskal-Wallis test was used to compare the flora structures of different households to obtain the 840 familial differential ASVs (*P* < 0.05) that distinguished them. These were used to create a cluster heat map that showed how most family members were clustered together. (c) Unweighted UniFrac distances in gut, oral, and skin flora structures between couples (C; gut, 35 pairs; oral, 39 pairs; skin, 49 pairs) and noncouples (NC; gut, 578 pairs; oral, 729 pairs; skin, 1,146 pairs), along with siblings (S; gut, 9 pairs; oral, 9 pairs; skin, 10 pairs) and nonsiblings (NS; gut, 72 pairs; oral, 72 pairs; skin, 90 pairs). Both pairs used the one-tailed Wilcoxon’s test for two single-factor comparisons (*, *P* < 0.05, ***, *P* < 0.001).

We further investigated the associations of microbiota compositions among subjects, both within and outside their families. Significant variations of microbiota among household microbiota were observed, regardless of sample location (oral: *R* = 0.27, *P* = 0.001; skin: *R* = 0.33, *P* = 0.001; gut: *R* = 0.33, *P* = 0.001) (Fig. 2a). Moreover, we performed similar analyses at the individual level by combining the amplicon sequence variants (ASVs) of the three ecological niches and observed consistent results (*R* = 0.40, *P* = 0.001; Fig. 2a). Meanwhile, cluster analysis clearly demonstrated higher degrees of similarity among the microbiota of family members than between unrelated individuals (Fig. 2b). These results demonstrate that flora structure is household-specific and exhibits high similarity within households.

Additionally, we investigated microbial composition associations between family members’ relationships, such as those between couples and siblings. We compared the similarities of gut, oral, and skin microorganisms between siblings and nonsiblings (also between couples and noncouples) based on unweighted UniFrac distance measurements. We found that the microbiota of couples and siblings were more similar to each other than those of noncouple and nonsibling pairs, which was likely due to alike living conditions, such as shared diets and companion times (Fig. 2c).

Overall, the above results indicate that the microbiota of family members are more similar than those of unrelated individuals, suggesting the significant roles of the family members in shaping children’s flora, which led us to further explore the patterns for microbial transmission between different adults and children within same house.

### Putative familial transmissible bacteria with diverse transmission ratios.

**FIG 3:**
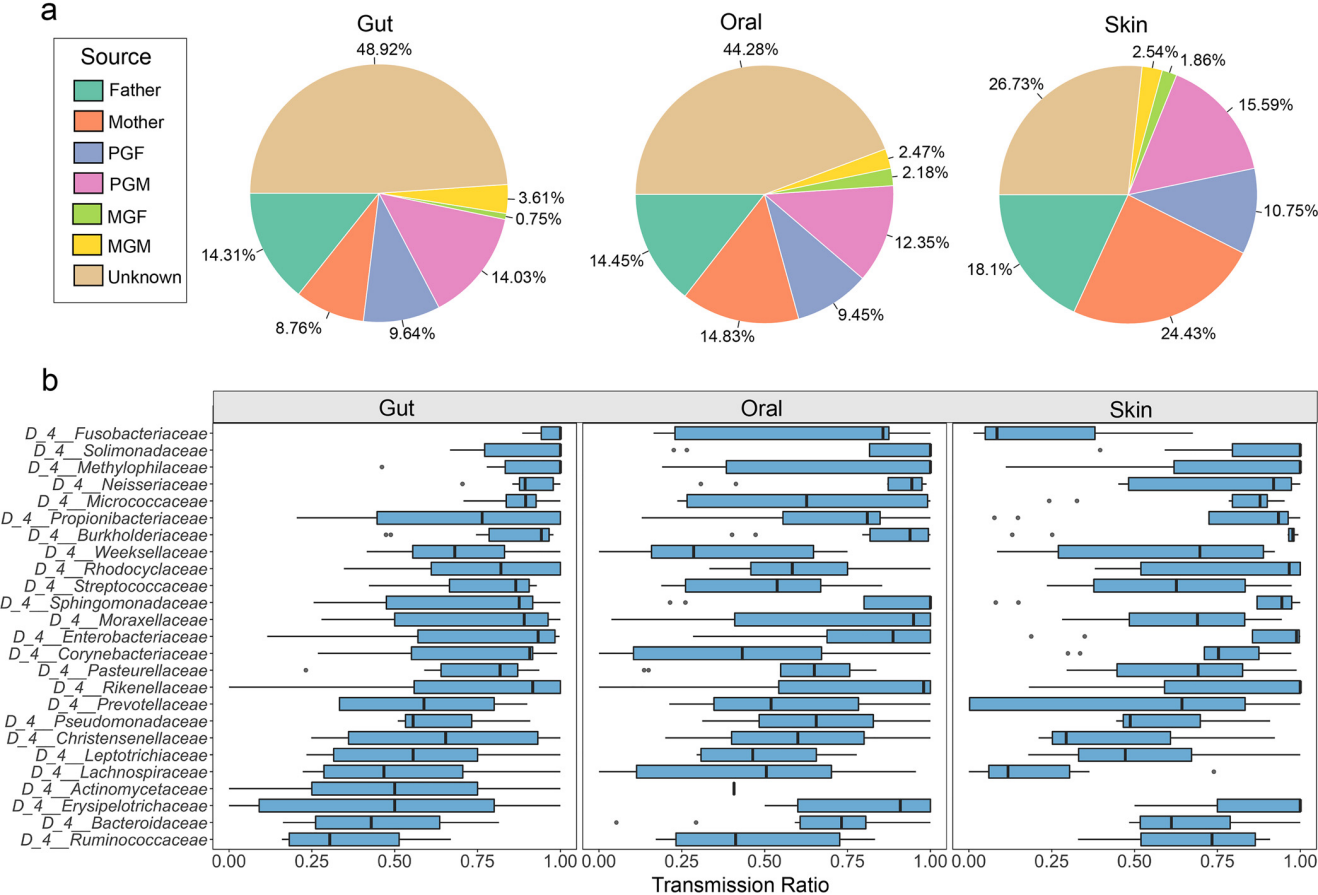
Sources of putative familial transmissible bacteria in the gut, oral, and skin flora of children. (a) Microbial source tracking results of gut, oral, and skin flora of children using FEAST, with percentages representing the contributions of each family member, as well as unknown sources of child flora. (b) Transmission of putative familial transmissible bacteria counted by FEAST. The *x* axis represents the transmission ratio (TR) of putative familial transmissible bacteria in different households.

Noting the high degree of microbial similarity between adults and children in the same family, we further performed microbiome source tracking analysis to focus on the role of family members in shaping children’s flora against the household-based cohort via fast expectation-maximization for microbial source tracking (FEAST) algorithm, which controlled the shared environmental confounders. More specifically, the microbiomes of children were set as sinks to estimate the proportions of their microbiomes that may have been derived from their family members. Microbial transmission proportion displayed no significant difference between different age groups or sex groups in this study, suggesting that the family members showed similar patterns on shaping the children microbiome regardless of children age and sex (Fig. S2 and S3). Generally speaking, over 50% of the children’s microbiota can trace their origins to the microbiota of parents and grandparents; in detail, an average of 76.73% of children’s skin microbiota was principally transmitted from their family members, as was 55.05% of children’s gut microbiota and 61.09% of their oral microbiota; moreover, mothers were observed to transmit a higher percentage of bacteria to their children compared to other family members, contributing to 8.05%, 13.03%, and 21.69% of their children’s gut, oral, and skin microbiota, respectively (Fig. 3a). We also compared the contributions of three niches of microbiota from each family member to children’s microbiome in Figure S4 and found that the mother’s oral and skin microbiota occupied transmission proportions significantly higher than those of other members. These results imply that the mother plays an important role in shaping of children’s microbiome, especially in mouth and skin.

Next, we focused on bacteria that can be traced from children to adults, where bacteria with transmission ratios (TRs) of >0 at the family level were designated “putative familial transmissible bacteria.” In total, there were 25 putative familial transmissible bacteria which were commonly detected in three ecological niches. These putative familial transmissible bacteria accounted for 17.36% of the gut microbiota at family level, along with 16.12% of oral microbiota and 4.23% of skin microbiota, respectively. However, the microbial transmission capabilities varied, meaning the bacteria displayed diverse TRs in different ecological niches of children (Table S1, Fig. 3b). For example, *Fusobacteriaceae* had high transmission capabilities in the gut (TR = 0.98) but was less transmitted in skin (TR = 0.44). TRs for *Moraxellaceae* in gut, oral, and skin were 0.75, 0.82, and 0.67, respectively. The pattern was similar for *Ruminococcaceae*, whose TR was 0.36 in gut and 0.47 in oral. In addition, the core familial transmissible bacteria differed among these three ecological niches. *Fusobacteriaceae* (TR = 0.98), *Solimonadaceae* (TR = 0.94), *Methylophilaceae* (TR = 0.92), *Neisseriaceae* (TR = 0.91), and *Micrococcaceae* (TR = 0.90) were the major putative familial transmissible bacteria in the gut, while *Methylophilaceae* (TR = 0.86), *Solimonadaceae* (TR = 0.85), and *Burkholderiaceae* (TR = 0.84) showed relatively higher transmission capabilities in the oral and *Solimonadaceae* (TR = 0.90) and *Erysipelotrichaceae* (TR = 0.87) were the core familial transmissible bacteria in the skin. Additionally, most putative familial transmissible bacteria were also familial differential ASVs (Fig. S5).

### Factors influencing putative familial transmissible bacteria.

Motivated by the existence of putative familial transmissible bacteria, we further explored potential factors driving the transfer of putative familial transmissible bacteria within households.

**FIG 4:**
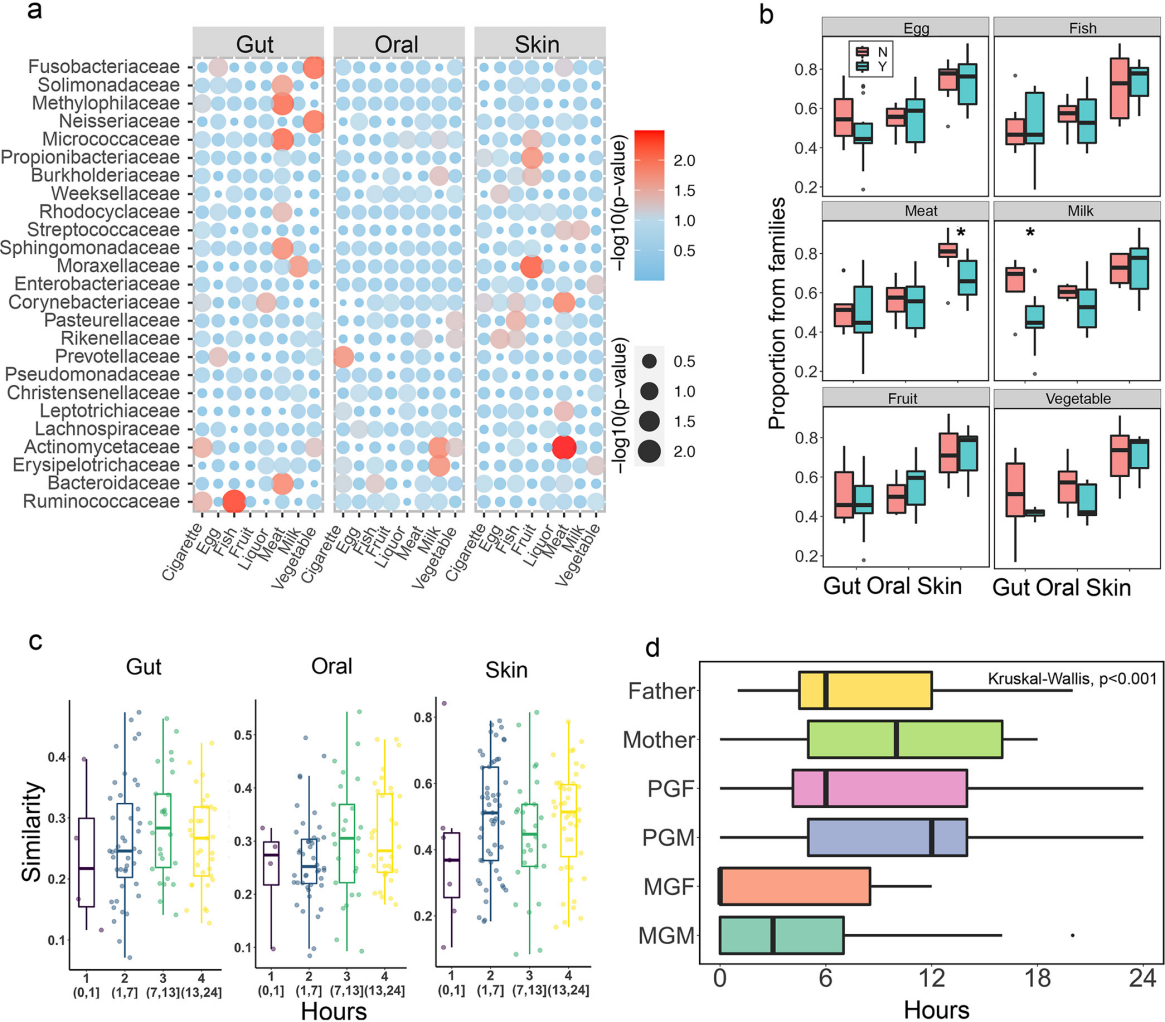
Influence of dietary preferences and family time on microbiome transmission. (a) The effect of dietary preferences on the abundance of putative familial transmissible bacteria in various parts of the children’s body. “Liquor” indicates whether alcohol is consumed in the child’s household and “cigarette” indicates whether smoking is present in the child’s household. Red represents the significant effect of a certain diet on the abundance of bacteria; the darker the color, the more significant the effect. Blue represents areas where the effect was not significant. (b) Proportion of flora coming from families, comparing children who do not ingest a certain food (N) to children who ingested a certain food (Y). *t* test, *, *P* < 0.05). (c) Similarity of flora between children and their family members was related to the time they spent together, where flora similarity was measured using the 1-Bray Curtis distance. (d) The time that child spent with each family member (in terms of hours per day) was recorded, showing that paternal grandmothers and mothers spent more time with their children than did others. The Wilcoxon’s test is used for global comparison of family time for each family member, while its pairwise test was presented in Figure S7c.

**Dietary preferences.** As dietary factors strongly influence microbiome composition (20), we explored the impact of dietary preferences on putative familial transmissible bacteria. Individuals who used antibiotics within the 3 months prior to the study were excluded from this analysis. Dietary preference factors included ingestion of fish, eggs, milk, meat, vegetables, fruits, liquor, and cigarettes, while the children’s liquor consumption or smoking was defined based on anyone in each household who smoked or drank alcohol. Both alpha and beta diversity analyses showed no significant differences in bacterial composition between individuals with and without specific dietary preferences (Fig. S6, Table S2). However, diet affects the abundance of the most putative familial transmissible bacteria, with intestinal flora affected most significantly (Fig. 4a, Fig. S7a and b). For children, some putative familial transmissible bacteria in the gut were more influenced by meat and vegetables, such as *Solimonadaceae*, *Methylophilaceae*, *Micrococcaceae*, *Fusobacteriaceae*, and *Sphingomonadaceae*. Cigarette, it should be noted, affected the *Actinomycetaceae* and *Ruminococcaceae* in children’s gut, followed by *Prevotellceae* in children’s oral. Although they do not smoke actively, family circumstances and family member interactions in family life may lead children to indirect intake. In addition, skin microbiota is more influenced by fruit and meat consumption, such as *Micrococcaceae*, *Propionibacteriaceae*, *Moraxellaceae*, along with *Corynebacteriaceae*, *Actinomycetaceae*, and *Streptococcaceae* (Fig. 4a). Interestingly, liquor has a greater influence on the putative familial transmissible bacteria in the male adults’ gut than on those in the female adults’ gut (Fig. S7a and b). These results indicate that transmittable bacteria are significantly affected by dietary preference. In addition, children with picky eating habits received a larger proportion of their microbial flora from their family members than nonpicky children did. In particular, we observed a significantly high proportion of bacteria from family members to skin microbiota of children who did not prefer meat and to gut microbiota in children who did not prefer milk, respectively (Fig. 4b). Therefore, the dietary habits of both children and adults can affect the transmission of flora between them.

**Family time.** Aside from dietary preference, we hypothesized that the amount of time that preceding generations spent with children also affected transmission. As expected, the microbial communities were more similar between family members and children as they spent more family time (FT) together (Fig. 4c). Moreover, among family members, parents and paternal grandparents spent significantly more time with their children/grandchildren than maternal grandparents, averaging 9.91 (mother [M]), 8.88 (father [F]), 9.38 (paternal grandfather [PGF]), 11.58 (paternal grandmother [PGM]), 5.3 (maternal grandfather [MGF]), and 3.9 (maternal grandmother [MGM]) hours of interaction per day, respectively (Fig. 4d, Fig. S7c). Accordingly, they contributed a larger proportion of flora to their children/grandchildren, especially skin flora, than maternal grandparents who spent less time with children (Fig. S4). This indicates that the time together may influence the transmission of flora between adult and child family members.

### The link between putative familial transmissible bacteria and children’s immune health.

**FIG 5:**
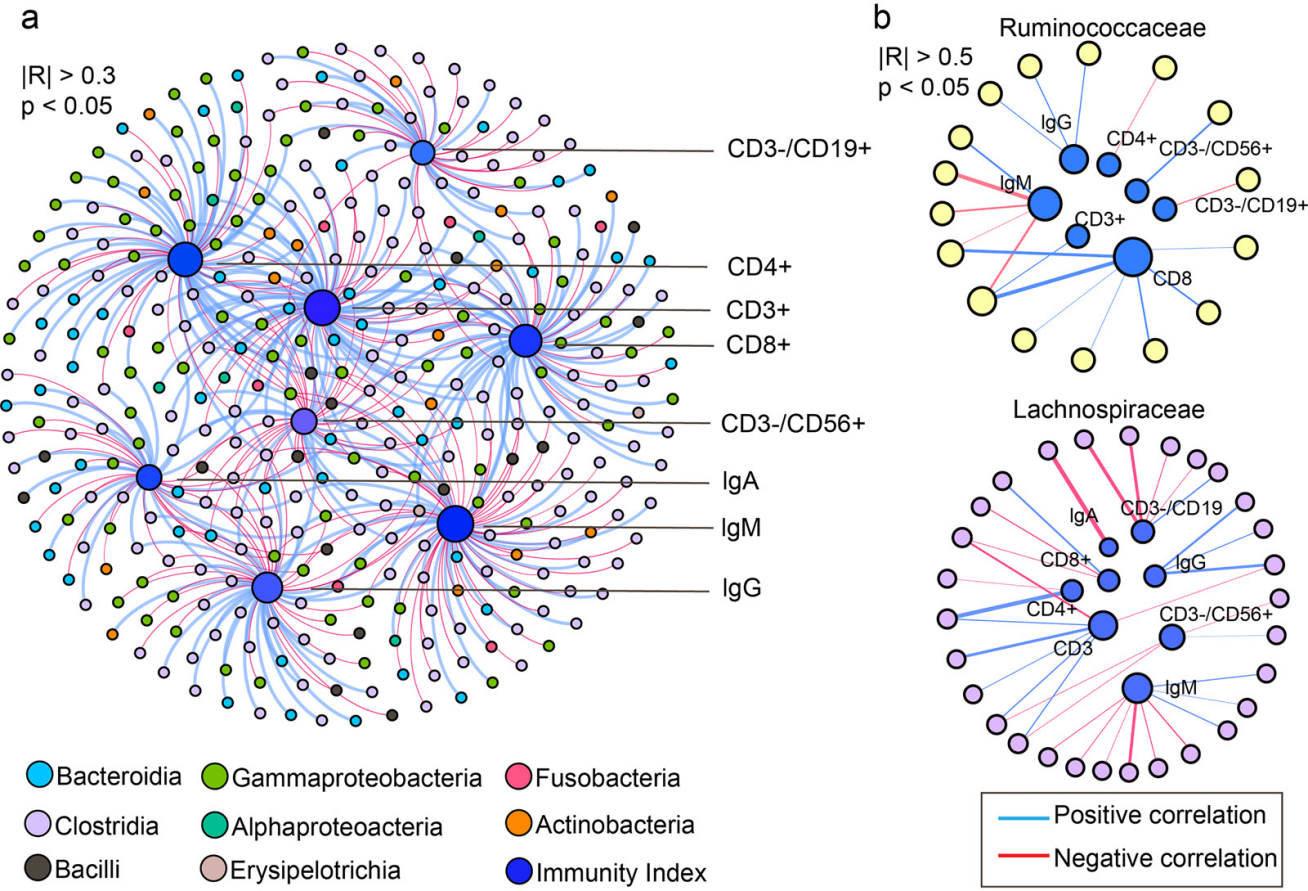
Transmissible flora linked to children’s immune health. (a) There are 353 ASVs correlated with immune indices (|R| > 0.3, *P* < 0.05) in the intestines of children, which were classified as transmissible bacteria. Their levels are labeled as classes. (b) *Ruminococcaceae* and *Lachnospiraceae* were highly correlated with the immune indices (|R| > 0.5). The blue line represents a positive correlation between ASVs and immune indices, whereas the red line represents a negative correlation. The thickness of the line indicates the strength of the correlation.

Considering the close link between immune responses and the gut microbiome, we further investigated the association between the gut microbiota and immune-related factors in children (Fig. 5). Immunoglobulins, including IgG, IgA, and IgM, and lymphocyte indices, including CD3^+^, CD4^+^, CD8^+^, CD3^−^/CD56^+^, and CD3^−^/CD19^+^, were considered. A total of 415 ASVs were found to be significantly associated with these immune markers. Of these, 353 (85.06%) ASVs were transmissible, indicating that putative familial transmissible bacteria may play an important role in childhood immunity. Among these 353 transmissible ASVs, 47.92% belonged to *Clostridia* (Table S3). IgM was strongly negatively associated with ASVs in the *Ruminococcaceae* and *Lachnospiraceae* families. ASVs in *Clostridia*, with a high correlation with immune indices, were mainly ASVs of *Ruminococcaceae* (*Subdoligranulum* sp., *Faecalibacterium* sp.), and *Lachnospiraceae* (*Lachnospiraceae NK4A136 group* sp., *Agathobacter* sp., *Coprococcus 2* sp.) (Table S3) showed a strong positive correlation (|R| > 0.5) with IgG. In addition, CD8^+^ was positively associated with *Ruminococcaceae* and negatively correlated with *Lachnospiraceae*.

## DISCUSSION

The majority of studies on the microbial transmission have focused on mothers’ role in shaping newborns, or infants’ microbiome (12, 13, 21). However, the microbial transmission stems from not only the mother’s physiological proximity but also from the overall social contact, shared environments in general, similar diets among family members, and host genetic influences (16, 22). Thus, the shaping of children’s microbiome is likely to be related to the transmission from their close family members, which cannot be ignored. In a recent study on Indian lineages, researchers explored microbiome compositions in three generations, focusing on the evaluation of age-associated microbiome changes. They identified core microbiome taxa by utilizing the Indian habit of living in multigenerational households, similar to living traditions in China, to control other causative factors behind microbiome composition (23). This method of controlling variables through cohort analysis on a household basis can be used to resolve the exact pattern of the shaping of children’s microbiome by family members within the same house. However, they did not focus on microbial transmission between generations.

In China, a similar traditional living mode means that parents, grandparents, and children spend more time together than they might otherwise, and dietary habits that involve a grouped dining system strengthen the relationship between generations. This allows researchers to better detect the relationships underlying microbiome transmission, providing an ideal research system for study. In this study, we analyzed the stool, oral, and skin microorganisms of 144 individuals from 24 families. Utilizing the unique living structures of Chinese family groups, we investigated patterns of microbiome transmission from older generations to genetically related children. Our results indicate that the impact of elderly family members on the formation of children’s microbiota has largely been underestimated. These also indicate that the inclusion of children’s family lives would be unavoidable subjects in the study of microbial colony shaping during early childhood.

It is reasonable that the microbiome composition is more similar among family members than between nonrelated individuals (Fig. 2), probably due to the shared environmental factors and the inherited factors. This is in line with previous studies showing that social networks were able to affect the composition of gut and oral microbiota (15–17, 24). Evidence gathered by examining the skin microbiomes of families over several weeks has shown that regular social interactions lead to more similar microbiome profiles among people, with microbiome compositions remaining distinct between different households (25). Meanwhile, we determined microbiome compositions in the gut, skin, and oral niches of three generations of our subjects’ families. The diversity indices and species richness of the gut microbiota in children were lower than those of the gut microbiota in adults (Fig. 1a), which also corresponded with the results of a previous study (26). Moreover, the microbiota of the three body sites sampled was more similar among relatives, such as couples and siblings, than among nonrelatives (Fig. 2c).

Although it has been verified that the microbiota can be transferred from mother to infant, the potential for sustained transmission after children reach 3 years of age remains unclear. However, long-term accumulation of the microbiota and changes in microbial composition remain important for children’s microbiomes. Therefore, we recruited children ranging from 0 to 12 years of age. In addition to infants, preschoolers (0 to 6 years old) and primary school students (over 6 years old) were also included in the effort to identify putative familial transmissible bacteria. Based on the data characteristics of household-based and child-centered, we identified family members as children microbiome’s potential origins and used the new resource trace software FEAST to trace the certain role played by adults in shaping the child’s microbiome. Using this, we established a model to predict the possible origin and transmission of microbiomes, which can capture more information on the basis of ASV level to effectively investigate the microbiome profile between children and their family members regardless of the taxonomy information of each ASV. Finally, we identified that 55.05% of the microorganisms in the intestinal flora of children originated from family members. In contrast, 76.73% of the microorganisms in the skin flora of children originated from family members (Fig. 3a). In addition, the family members showed similar patterns in shaping the children microbiome regardless of children age and sex (Fig. S2 and S3). Mothers transmitted a higher percentage of bacteria to children compared to other family members (8.05%, 13.03%, and 21.69% of gut, oral, and skin microbiota, respectively) (Fig. 3a, Fig. S4), suggesting that the mother plays the important role in shaping the flora of the growing child, not only in the delivery process. These results indicate that the transmission of microbiota from family members to children exactly occurs in a three-generation household and the transmission of family flora plays a significant role in shaping the flora of children.

A total of 25 putative familial transmissible bacteria (at family level) were identified in our study, and some of them were also found to be family core bacteria in others studies (23), such as *Neisseriaceae* (TR = 0.83), *Fusobacteriaceae* (TR = 0.63), and *Burkholderiaceae* (TR = 0.84) in the mouth, *Streptococcaceae* (TR = 0.60) and *Corynebacteriaceae* (TR = 0.71) in the skin, and *Prevotellaceae* (TR = 0.71) in the gut. It has been reported that these major familial transmissible bacteria also possess important biofunctions for human health or disease. Taxa belonging to *Neisseriaceae* could produce γ-glutamyl-transferase, which has been widely applied for clinic diagnosis, such as fatty liver, infectious hepatitis, cardiovascular disease, and pancreatitis (27–29). *Fusobacterium* has been widely reported to be involved with colorectal carcinoma, periodontal diseases, and Lemierre’s syndrome (30–32). *Streptococcaceae* was detected with enriched abundance in patients with ulcerative colitis (UC), acute-on-chronic liver failure, and even pediatric patients with chronic serous otitis media, while the abundance of *Prevotellaceae* was depleted in UC patients (33–35). Thus, the roles of these putative familial transmissible bacteria could not be ignored.

Many reports have shown that diet contributes to the maturation of infant gut microbiota and dietary alterations can induce microbiome associated changes (20, 36, 37). This might be a significant factor in microbial differences between children and adults. In addition, children’s microbiomes might be unstable owing to their lower level of exposure to the outside environment, which could be another factor differentiating children from adults. There was some correlation between children’s dietary habits and gut microbial composition in some studies; for example, ingestion of non-whole-grain foods and a high intake of dietary fructose could affect gut microbial composition in children (38, 39). The results of this study were consistent with previous reports that the dietary preferences of children influence microbiome composition (Fig. 4a). Children with fussy diets tended to receive a larger proportion of their microbial flora from their family members than from the environment (Fig. 4b), indicating that a healthy and balanced diet will be beneficial in increasing sources of colonization of children’s intestinal flora, potentially increasing their flora species. In addition, there are more putative familial transmissible bacteria significantly affecting adults than those affecting children, which may be attributed to a longer period of time to develop this habit and the direct intake in adults (Fig. S7a and b).

Furthermore, we found that the more family time the members spend with children, the more similar the microbial communities are, though this acheived no statistical significance (Fig. 4c). Consistently, the children traced significantly less of their bacteria from maternal grandparents among all family members, which is possibly related to less family time (Fig. 4d, Fig. S4, Fig. S7c). Therefore, family members should be warned that their lifestyles are equally important for shaping children’s healthy flora. Since this is a proof-of-concept study, more evidence with large sample size and controlling for variables more accurately is needed.

Moreover, we explored associations between the putative familial transmissible bacteria and immune system development in children. A total of 353 ASVs belonging to putative familial transmissible bacteria were found to be significantly associated with immune indicators (Fig. 5, Table S3). At the same time, most putative familial transmissible bacteria exhibited different abundances in different households (Fig. S5, Table S3). This shows that putative familial transmissible bacteria can produce differences between the flora of different households, which are in relation to immune health. Transmittable ASVs belonging to *Ruminococcaceae* and *Lachnospiraceae*, in particular, were strongly correlated with immune indicators, such as CD8^+^, CD3^+^, IgG, and IgM. It is well known that *Ruminococcaceae* and *Lachnospiraceae* are able to produce beneficial metabolites for the host, such as butyrate and other short chain fatty acids (SCFAs) (40–42). The change in abundance of *Lachnospiraceae* might be related to metabolic disease, primary sclerosing cholangitis, and/or Crohn’s disease (43–45). Therefore, it is better to utilize metagenomic sequencing rather than 16S rRNA sequencing to analyze the functions of *Lachnospiraceae* in immunity, which are subjects of interest for further study.

We acknowledge shortcomings of our study, such as that the sample size was not large enough, especially the number of children. Since this is a proof-of-concept study, research with large-scale samples on microbiome pedigree including three generations in China could be a further target. Another imperfection is that we chose only one sampling site for skin microbiome. Several body sites were used as sampling site for skin microbiome described in some research literatures, such as glabella, antecubital fossa, volar forearm, toe web space, nose, and so on (46–48). Considering that skin swab sampling on antecubital fossa is more convenient for children and this site could make contact with family members or living environments appropriately, we selected antecubital fossa as the sampling site, but the limitation is that the status of skin microbiome is not comprehensive to exhibit.

### Conclusions.

In summary, the present study reveals that children’s microbiomes can be transmitted from their family members, including those other than their mothers. It also proposes the concept of putative familial transmissible bacteria, quantifying microbiome transmission between children and their families. Moreover, we demonstrated that family time and dietary preferences of children are important factors that affect putative familial transmissible bacteria, which then play critical roles in immune system development. This study highlights the importance of home life in the early development of children’s microbiomes and provides potential strategies for shaping healthy microbiomes during childhood.

## MATERIALS AND METHODS

### Participants.

This study was approved by the Ethics Committee of the Qilu Children’s Hospital, affiliated with the Cheeloo College of Medicine of Shandong University (Jinan Children’s Hospital, No. QLET-IRB/T-2021001).

The recruited participants included staff at Qilu Children’s Hospital and three generations of their family members. A total of 144 individuals from 24 families were enrolled in this study, with families with inherited diseases excluded from participation. All participants provided written informed consent.

### Information and sample collection.

Eligible participants self-reported information deemed relevant to the study. This information was collected using a questionnaire that asked about their basic physical conditions, current age, hours spent interacting with children, and dietary preferences.

Fecal samples were collected as described previously (49). Saliva samples (approximately 5 mL) were collected in sterile tubes. Skin swab samples were obtained using sterile cotton swabs soaked in sterile saline solution containing 0.1% Tween 20. Swabs were gently rubbed across 5 cm^2^ areas on the participants’ antecubital fossa regions, where skin could make contact with others and is convenient to access especially for children, inserted into tubes containing bacterial storage solution, and vortexed vigorously for 30 s. Venous blood samples were obtained from the hospital following the rules of clinical examination. All the samples were frozen at −80°C until they were subjected to further processing.

### Immune indices examination.

Concentrations of human immunoglobulins (IgA, IgG, and IgM) in serum were examined via rate nephelometry, using the SIEMENS BNII ProSpec system. Lymphocyte indices (CD3^+^, CD4^+^, CD8^+^, CD3^−^/CD56^+^, and CD3^−^/CD19^+^) of whole blood were determined via flow cytometry, using a Beckman FC500. All procedures were performed according to operating instructions.

### Microbiota 16S amplicon sequencing.

Frozen stool samples were processed for DNA extraction using the QIAamp PowerFecal DNA kit, following the manufacturer’s instructions. Total bacterial DNA extraction from saliva and skin swab samples was performed according to the QIAamp BIOstic Bacteremia DNA kit manufacturer’s instructions. The hypervariable V1-V2 region of the 16S rRNA gene was amplified by PCR, using forward primer 5′-XXXXXXAGAGTTTGATCCCTGGCTCAG-3′ and reverse primer 5′-XXXXXXTGCTGCCTCCCGTAGGAGT-3′ (50), where X represents a barcode base. PCR products were quantified via gel electrophoresis, purified using the QIAquick PCR purification kit, and quantified using a NanoDrop ND2000 spectrophotometer. The constructed DNA libraries were sequenced on an Illumina HiSeq 2500 platform with 2 × 250 bp paired-end reads. All data can be viewed in NODE (http://www.biosino.org/node) via the accession OEP000631 or through the URL http://www.biosino.org/node/project/detail/OEP000631.

### Bioinformatic analysis of 16S rRNA gene sequencing data.

16S rRNA sequencing data were processed using the Quantitative Insights Into Microbial Ecology 2 (QIIME2 V.2019.07) platform (51). Primer sequences and barcode sequences were first removed using Cutadapt (52). Then, DADA2 software (53), wrapped in QIIME2, was used to filter out low-quality sequencing reads (Q < 30). High-quality reads were denoised into amplicon sequence variants (ASVs, 100% exact sequence match), resulting in an ASV count table and representative sequences. Representative sequences of each ASV were aligned via Fast Fourier Transform in Multiple Alignment (MAFFT) (54) in the q2-phylogeny plugin, and a phylogenetic tree was constructed using the Fast-Tree plugin (55). Finally, the taxonomies of ASVs were assigned via Naïve Bayes classifier (56) trained on 99% clustered sequences in the Silva-132-99 reference database (57). The feature count table was then converted into several relative abundance tables for further analysis. Alpha and beta diversities were calculated based on the rarefied count table, at a depth of 7,115 reads.

### Microbial source tracking analysis.

Fast expectation-maximization for microbial source tracking (FEAST) (58) is a tool that estimates the proportions of the microbial community in the sink, which was derived from the potential source environment. This was then used to quantify the proportion of children’s microbiota (sink) that was contributed by the potential origins from every family member (source). In detail, FEAST takes the profile of family members’ microbiome *X*_member_ as input, which also contains the shared environmental confounders, and assumes each sink is a convex combination of known and unknown sources; thus, the children’s microbiome can be given by

$$X_{C}\;=\;\alpha_{1}X_{F}\;+\;\alpha_{2}X_{M}\;+\;\alpha_{3}X_{\mathrm{PGM}}\;+\;\alpha_{4}X_{\mathrm{PGF}}\;+\;\alpha_{5}X_{\mathrm{MGM}}\;+\;\alpha_{6}X_{\mathrm{MGF}}\;+\;\alpha_{7}\mathrm{Unknown}$$

where

$$\overset{7}{\underset{i=1}{\sum }}\alpha_{i}\mathrm{= 1}$$

The α*_i_* would be evaluated by expectation-maximization algorithm, indicating the proportion of the sink community that was contributed by each source environment. The *X*_member_ is the profile of each family member’s microbiome at ASV level.

We also calculated the quantity of bacteria that could be transmitted within the household at the bacteria family level. In addition, the transmission ratio (TR) was defined as the proportion of a particular bacterium from family members that is transmitted to that family’s children. This allowed us to describe microbiome transmission capability. Bacteria with a TR of >0 were considered “putative familial transmissible bacteria.”

### Statistical analysis.

Unweighted UniFrac distance (59) was used to estimate beta diversity, which was visualized using principal coordinate analysis (PCoA) plots.

To investigate microbiota composition associations among subjects, both inside and outside their households, we compared a household as a unit. Analysis of similarities (ANOSIM), based on the Bray Curtis distance, was performed using vegan package to measure the similarity in gut, oral, skin, and total individual microbial compositions between members of different households (60). We also launched global and pairwise comparisons of the microbiome structures of each ecological niche between members of each household. To provide a comprehensive picture of individual microbiome composition, we combined the ASVs from the three ecological niches on the individual level as well.

Familial differential ASVs (*P* < 0.05) were identified using one-way analysis of variance on ranks (Kruskal-Wallis test) on individual units. Spearman correlations (|r| > 0.3, *P* < 0.05) were performed to explore potential relationships among family members based on relative abundance at the ASV level via the “corr” function in the corrplot package (61). Correlations were visualized using the “pheatmap” package (62). Furthermore, associations between immune index and transmissible ASVs in children were calculated using the “rcorr” function in the Hmisc package and were displayed via Gephi (63). All statistical analyses in this study were performed in the R environment (version 4.0.2), most of which used the vegan package (60). Other plots were generated using the ggplot2 package (64).

### Data availability.

All sequencing data were uploaded to NODE (accession no. OEP00631) and can be accessed at http://www.biosino.org/node.
