# Supplementary material for: Putative Familial Transmissible Bacteria of Various Body Niches Link with Home Environment and Children’s Immune Health
Source: Microbiol Spectr. 2021 Dec 8;9(3):e00872-21. doi: 10.1128/Spectrum.00872-21 (PMC8653841; doi:10.1128/Spectrum.00872-21)
Supplement: SUPPLEMENTAL FILE 1 — Supplemental material. Download SPECTRUM00872-21_Supp_1_seq10.pdf, PDF file, 1.6 MB [file spectrum00872-21_supp_1_seq10.pdf]

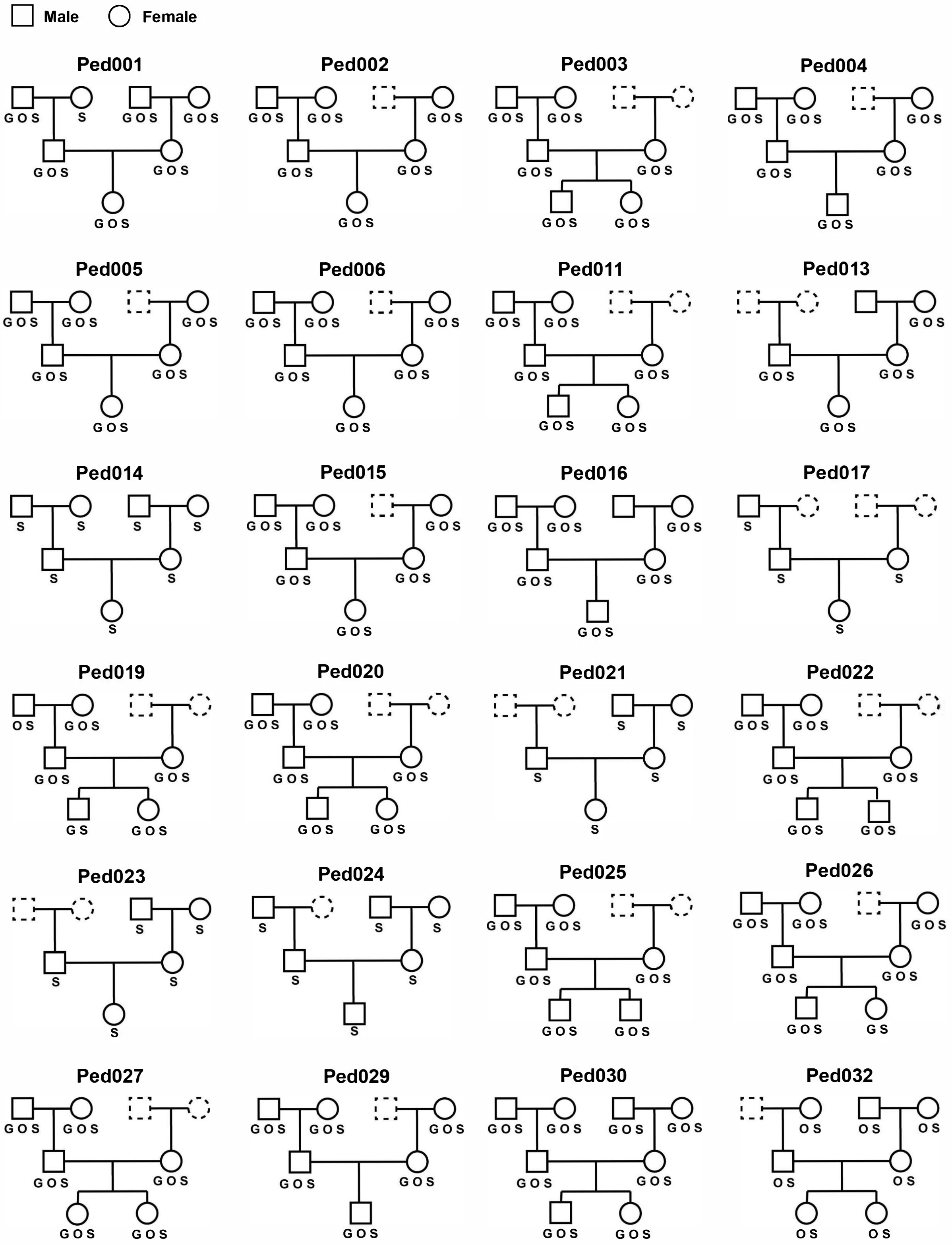
Male
Female
Ped001
Ped002
Ped003
Ped004
Ped005
Ped006
Ped011
Ped013
Ped014
Ped015
Ped016
Ped017
Ped019
Ped020
Ped021
Ped022
Ped023
Ped024
Ped025
Ped026
Ped027
Ped029
Ped030
Ped032

Figure S1 Familial status of the samples. Shows the relatives of the sample households, where the dashed box represents the sample of people who was absent. ‘G’,’O’,’S’ means the ecological niches, while ‘G’ for gut, ‘O’ for oral and ‘S’ for skin.

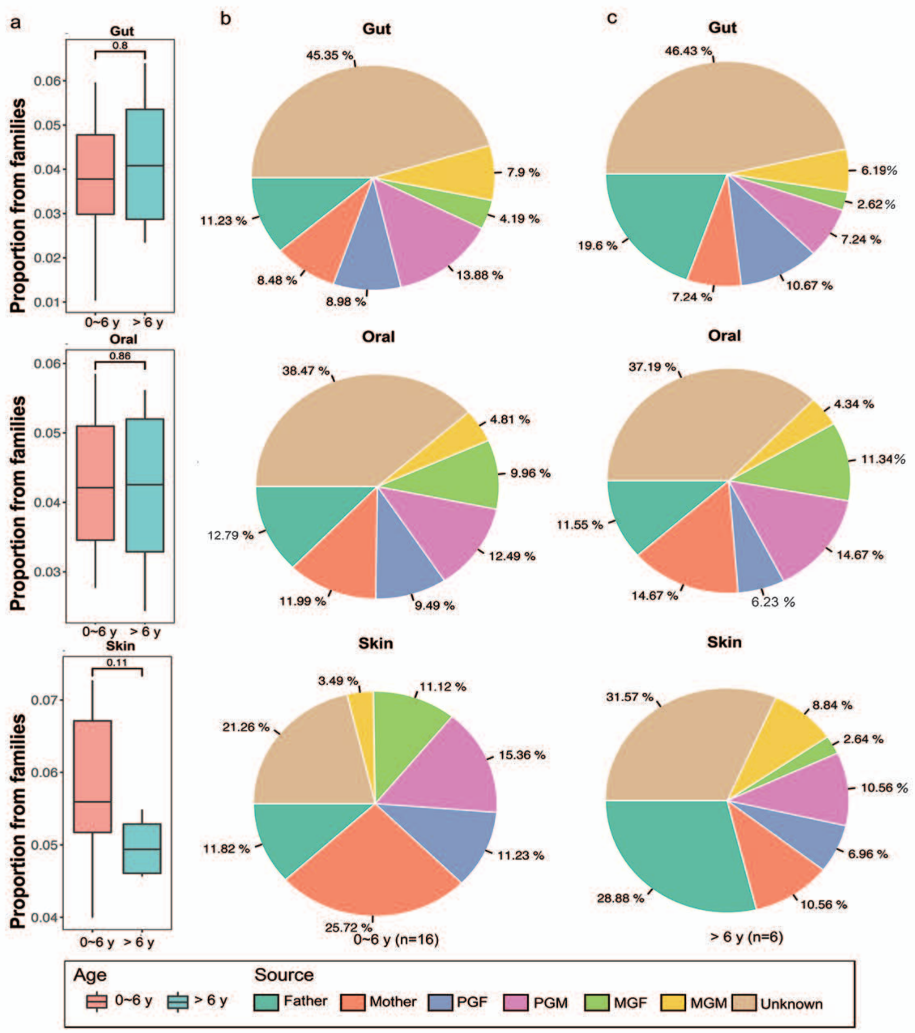

a
b
c
Gut
Oral
Skin
Proportion from families
0.8
0.86
0.11
0~6 y
> 6 y
45.35 %
7.9 %
4.19 %
13.88 %
8.98 %
8.48 %
11.23 %
46.43 %
6.19%
2.62 %
7.24 %
10.67 %
7.24 %
19.6 %
38.47 %
4.81 %
9.96 %
12.49 %
9.49 %
11.99 %
12.79 %
37.19 %
4.34 %
11.34%
14.67 %
6.23 %
14.67 %
11.55 %
3.49 %
11.12 %
15.36 %
11.23 %
25.72 %
11.82 %
21.26 %
31.57 %
8.84 %
2.64 %
10.56 %
6.96 %
10.56 %
28.88 %
0~6 y (n=16)
> 6 y (n=6)
Age
0~6 y
> 6 y
Source
Father
Mother
PGF
PGM
MGF
MGM
Unknown

Figure S2 Microbial source tracking results of gut, oral, and skin flora of children of different ages using FEAST. Considering the Chinese education policy, we divided the samples of children into two groups, preschoolers (0-6 years old) and primary school-children (over 6 years old), by using the age of six as cut-off. What's more, the main life environment of preschoolers is their own family, which has more frequent contact with their family members. In contrast, primary school children spend more time in school, which may contact with others besides family members. (a) Proportion of contribution of each ecological niche of each family member to children's gut, oral and skin flora. Wilcoxon test was used to compare the difference between groups, and the P-values were labeled. (b) Pie Charts with percentages representing the contributions of each family member to preschoolers' gut, oral and skin flora, as well as unknown sources of their flora. (c) Pie Charts with percentages representing the contributions of each family member to primary school children's gut, oral and skin flora, as well as unknown sources of their flora.

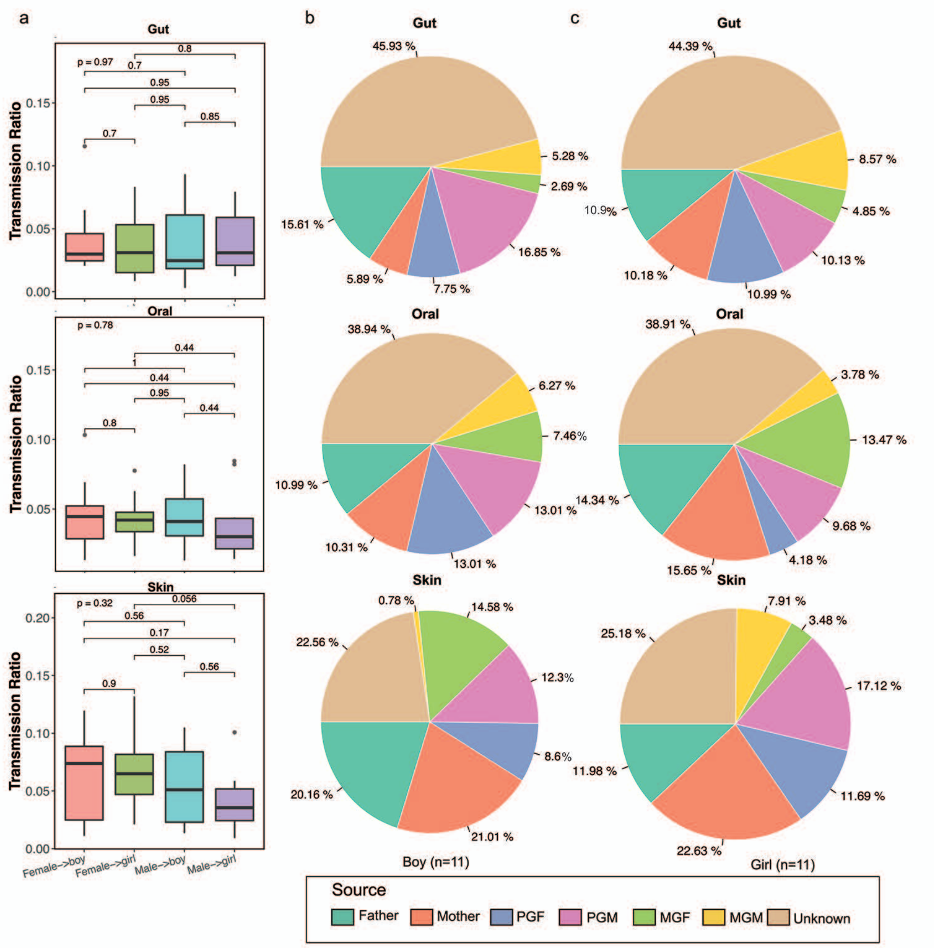
a
b
c
Gut
Oral
Skin
Transmission Ratio
Female->boy
Female->girl
Male->boy
Male->girl
Boy (n=11)
Girl (n=11)
Source
Father
Mother
PGF
PGM
MGF
MGM
Unknown

Figure S3 Microbial source tracking results of gut, oral, and skin flora of children of different gender using FEAST. (a) Proportion of contribution of each ecological niche of each family member to children's gut, oral and skin flora. Wilcoxon test was used to compare the difference between groups, and the P-values were labeled. (b) Pie Charts with percentages representing the contributions of each family member to boys' gut, oral and skin flora, as well as unknown sources of their flora. (c) Pie Charts with percentages representing the contributions of each family member to girls' gut, oral and skin flora, as well as unknown sources of their flora.

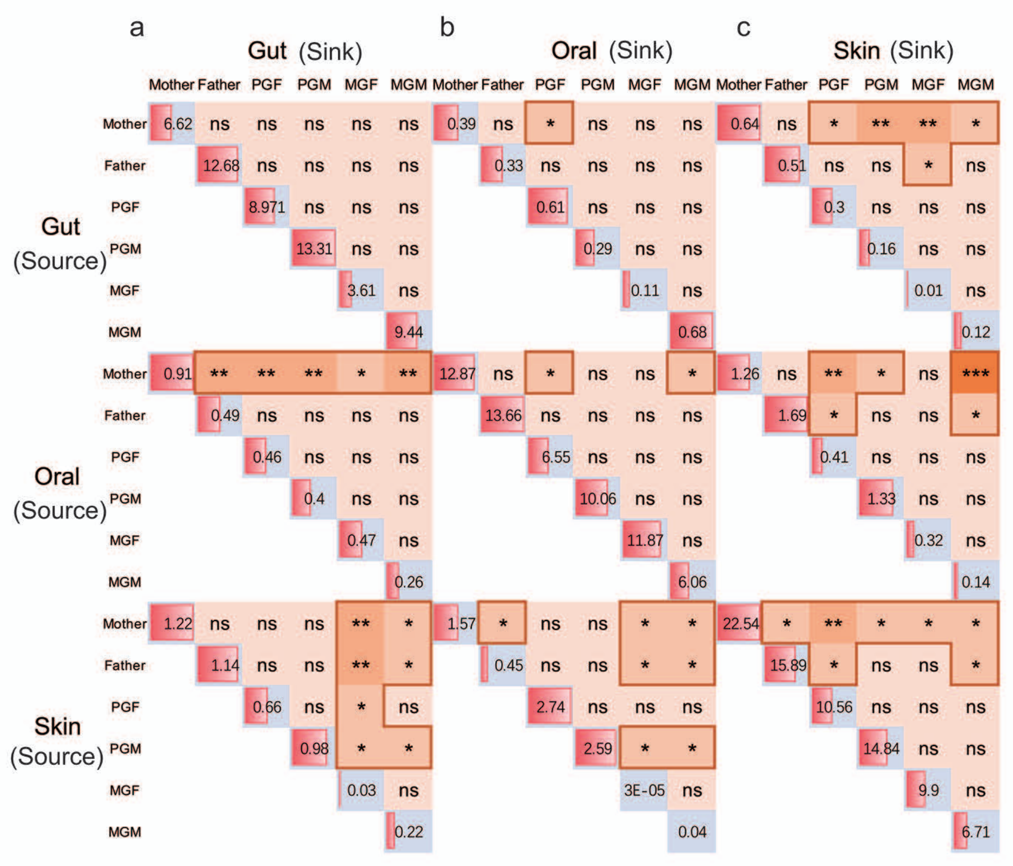
a
Gut (Sink)
b
Oral (Sink)
c
Skin (Sink)
Mother Father PGF PGM MGF MGM Mother Father PGF PGM MGF MGM Mother Father PGF PGM MGF MGM
Gut (Source)
Mother 6.62 ns ns ns ns ns 0.39 ns * ns ns ns 0.64 ns * ** ** *
Father 12.68 ns ns ns ns 0.33 ns ns ns ns 0.51 ns ns * ns
PGF 8.971 ns ns ns 0.61 ns ns ns 0.3 ns ns ns
PGM 13.31 ns ns 0.29 ns ns 0.16 ns ns
MGF 3.61 ns 0.11 ns 0.01 ns
MGM 9.44 0.68 0.12
Oral (Source)
Mother 0.91 ** ** ** * ** 12.87 ns * ns ns * 1.26 ns ** * ns ***
Father 0.49 ns ns ns ns 13.66 ns ns ns ns 1.69 * ns ns *
PGF 0.46 ns ns ns 6.55 ns ns ns 0.41 ns ns ns
PGM 0.4 ns ns 10.06 ns ns 1.33 ns ns
MGF 0.47 ns 11.87 ns 0.32 ns
MGM 0.26 6.06 0.14
Skin (Source)
Mother 1.22 ns ns ns ** * 1.57 * ns ns * * 22.54 * ** * * *
Father 1.14 ns ns ** * 0.45 ns ns * * 15.89 * ns ns *
PGF 0.66 ns * ns 2.74 ns ns ns 10.56 ns ns ns
PGM 0.98 * * 2.59 * * 14.84 ns ns
MGF 0.03 ns 3E-05 ns 9.9 ns
MGM 0.22 0.04 6.71

Figure S4 Comparison of the differences in the contribution of the gut, oral and skin microbiota of each family member to the gut (a), oral (b) and skin (c) microbiota of the children. The average microbial transmission proportions of the sources are marked on the diagonal. Among them, the contribution of the mother's flora to the child was significantly higher than that of other family members in almost every niche. Wilcoxon's test, *:$p<0.05$,**:$p<0.01$, ***: $p<0.001$.

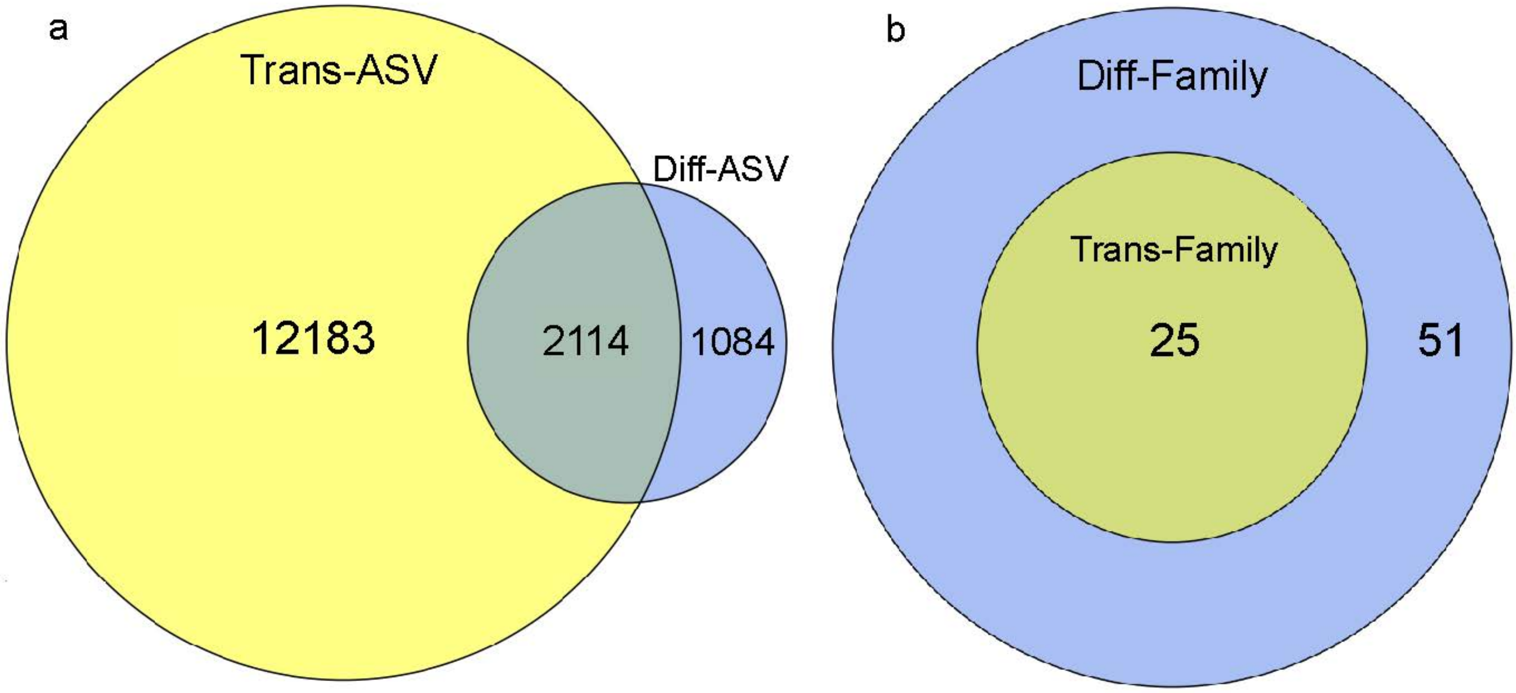
a
Trans-ASV
Diff-ASV
12183
2114
1084
b
Diff-Family
Trans-Family
25
51

Figure S5 The sharing between transferable bacteria (Trans-ASV/Trans-Family) and family differential bacteria (Diff-ASV/Diff-Family). Diff-ASV/Diff-Family ($p<0.05$) were differential microbiome between households in ASV/Family taxonomy level identified by one-way ANOVA. Trans-ASV/Trans-Family were microbiomes that can transmit in households identified by FEAST($TR>0$) in ASV/Family taxonomy level. (a) Most of the differential ASVs are transmissible in the household;(b) All transmissible families are differential families.

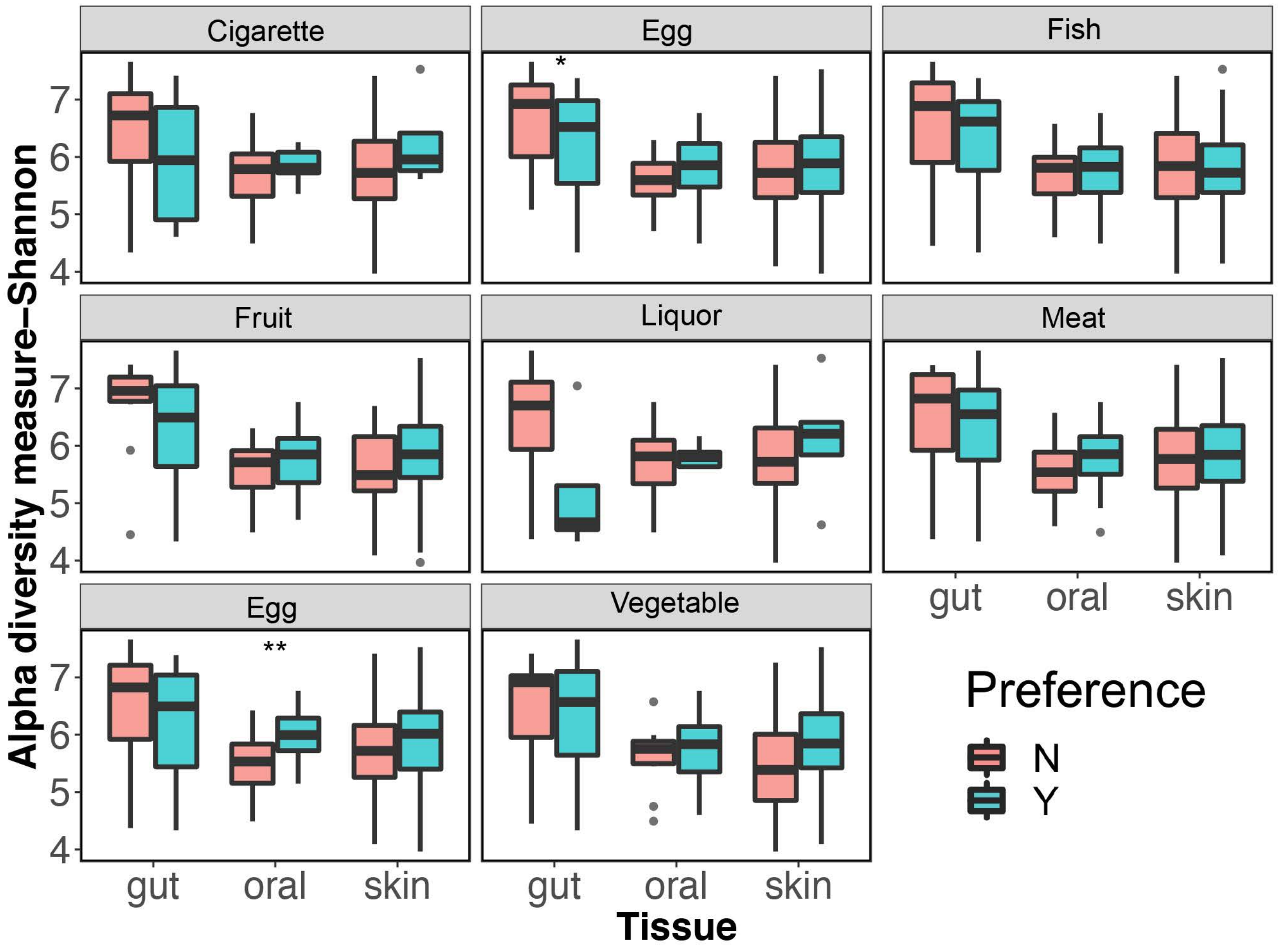

Cigarette
Egg
Fish
Fruit
Liquor
Meat
Egg
Vegetable
*
**
Alpha diversity measure−Shannon
4
5
6
7
gut
oral
skin
Tissue
Preference
N
Y

Figure S6 Alpha diversity of flora in populations with different dietary preferences. Dietary habits have an impact on the diversity of human flora. Some of the data in this study were not significant, which may be related to the small sample size. N: people who would not eat certain food, Y: people who would like to eat certain food. Wilcoxon's test, *:$p<0.05$,**:$p<0.01$, ***: $p<0.001$.

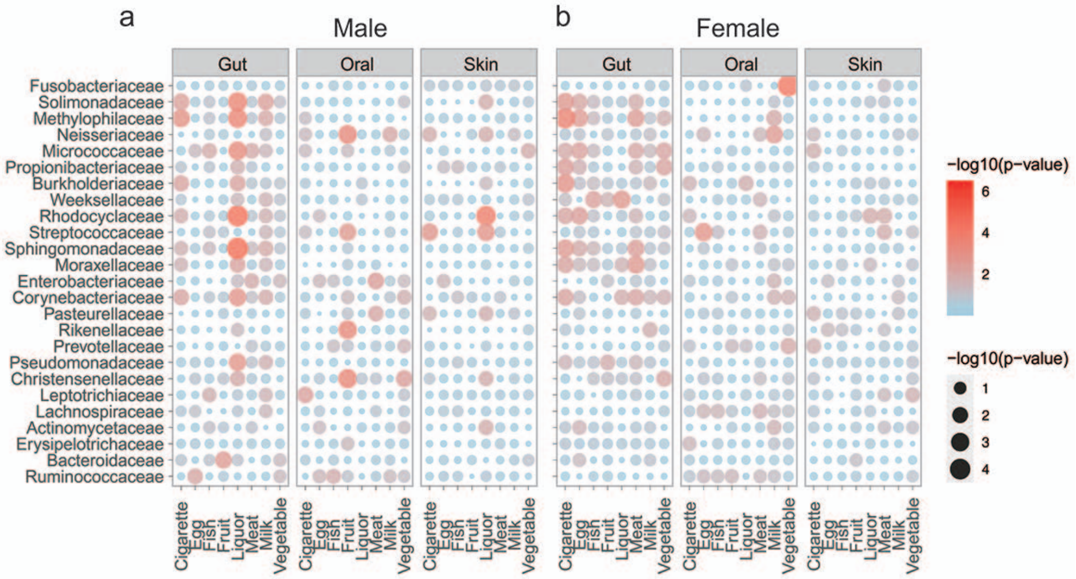

c

Family time(h/d)		Mother	Father	PGF	PGM	MGF	MGM
9.9	Mother		ns	ns	ns	***	****
8.7	Father	-		ns	ns	***	***
9.4	PGF	-	-		ns	*	ns
11.6	PGM	-	-	-		***	***
5.3	MGF	-	-	-	-		ns
3.9	MGM	-	-	-	-	-	

Figure S7 Influence of dietary preferences on putative familial transmissible bacteria of adults and comparison of differences in family time of family members. (a-b)The effect of dietary preferences on the abundance of putative familial transmissible bacteria in various parts of the males' body(a) or females' body(b). Individuals who used antibiotics within the three months prior to the study were excluded. Since there are no females smoking in this dataset, where "Cigarette" in (b) indicates whether smoking is present in her household. Red represents the significant effect of a certain diet on the abundance of bacteria (Wilcoxon's test); the darker the colour, the more significant the effect. Blue represents areas where the effect was not significant. (c) Comparison of the distribution differences in the family time(in terms of hours per day) between family members, the bar chart shows the average hours children spent with each family member. Wilcoxon's test, *:$p<0.05$,**:$p<0.01$, ***: $p<0.001$.

Supplementary Table 1*

**Family(taxonomic rank)**	**Father**	**Mother**	**PGF**	**PGM**	**MGF**	**MGM**	**Unknown**
Actinomycetaceae	0.46±0.41	0.00±0.00	0.01±0.00	0.00±0.00	0.00±0.00	0.03±0.00	0.50±0.50
Bacteroidaceae	0.11±0.03	0.09±0.01	0.12±0.02	0.05±0.00	0.01±0.00	0.07±0.01	0.55±0.06
Burkholderiaceae	0.16±0.03	0.35±0.06	0.13±0.02	0.08±0.02	0.00±0.00	0.09±0.03	0.16±0.04
Christensenellaceae	0.07±0.01	0.38±0.11	0.15±0.06	0.03±0.00	0.00±0.00	0.00±0.00	0.36±0.14
Corynebacteriaceae	0.11±0.01	0.23±0.05	0.12±0.01	0.14±0.02	0.04±0.02	0.08±0.01	0.26±0.08
Enterobacteriaceae	0.24±0.05	0.18±0.04	0.05±0.00	0.2±0.04	0.00±0.00	0.07±0.01	0.26±0.12
Erysipelotrichaceae	0.05±0.01	0.25±0.06	0.00±0.00	0.1±0.02	0.00±0.00	0.04±0.01	0.52±0.19
Fusobacteriaceae	0.35±0.07	0.13±0.02	0.21±0.05	0.11±0.02	0.00±0.00	0.18±0.03	0.02±0.00
Lachnospiraceae	0.08±0.01	0.09±0.02	0.13±0.03	0.16±0.04	0.02±0.00	0.03±0.01	0.47±0.09
Leptotrichiaceae	0.04±0.01	0.16±0.08	0.04±0.00	0.15±0.11	0.00±0.00	0.14±0.06	0.43±0.10
Methylophilaceae	0.19±0.00	0.25±0.01	0.15±0.01	0.15±0.00	0.05±0.01	0.12±0.01	0.08±0.03
Micrococcaceae	0.1±0.01	0.25±0.07	0.25±0.06	0.13±0.04	0.00±0.00	0.15±0.07	0.10±0.01
Moraxellaceae	0.14±0.02	0.27±0.06	0.07±0.00	0.14±0.02	0.05±0.02	0.08±0.02	0.25±0.08
Neisseriaceae	0.34±0.1	0.12±0.02	0.21±0.11	0.16±0.05	0.02±0.00	0.06±0.01	0.09±0.01
Pasteurellaceae	0.21±0.05	0.09±0.02	0.13±0.02	0.12±0.01	0.01±0.00	0.15±0.07	0.26±0.04
Prevotellaceae	0.1±0.05	0.22±0.04	0.07±0.00	0.1.±0.02	0.08±0.03	0.15±0.02	0.29±0.09
Propionibacteriaceae	0.23±0.06	0.22±0.05	0.07±0.01	0.1±0.01	0.02±0.00	0.23±0.08	0.13±0.08
Pseudomonadaceae	0.07±0.01	0.19±0.01	0.13±0.01	0.19±0.03	0.00±0.00	0.08±0.02	0.34±0.05
Rhodocyclaceae	0.26±0.05	0.12±0.01	0.15±0.03	0.17±0.02	0.00±0.00	0.07±0.03	0.20±0.07
Rikenellaceae	0.08±0.02	0.08±0.01	0.23±0.13	0.09±0.04	0.02±0.00	0.21±0.07	0.28±0.17
Ruminococcaceae	0.05±0.00	0.13±0.03	0.08±0.01	0.06±0.01	0.02±0.00	0.02±0.00	0.64±0.04
Solimonadaceae	0.23±0.10	0.26±0.12	0.07±0.01	0.11±0.01	0.03±0.01	0.22±0.12	0.06±0.02
Sphingomonadaceae	0.13±0.06	0.12±0.03	0.20±0.08	0.16±0.07	0.02±0.00	0.15±0.08	0.23±0.08
Streptococcaceae	0.22±0.07	0.14±0.06	0.20±0.07	0.04±0.00	0.04±0.02	0.13±0.07	0.22±0.03
Weeksellaceae	0.18±0.05	0.19±0.07	0.08±0.02	0.24±0.12	0.02±0.00	0.12±0.05	0.18±0.05

*Average transmission ratio of microbe (%). Data are the mean ± SD.
The list of familial transmissible bacteria at the family level, which were identified by FEAST(TR>0). All familial transmissible bacteria contain household differential ASVs.

Supplementary Table 2

	Gut		Oral		Skin	
	**R**	**p-value**	**R**	**p-value**	**R**	**p-value**
Fish	-0.035	0.71	-0.018	0.62	0.005	0.33
Egg	-0.033	0.75	-0.022	0.64	0.000	0.39
Milk	0.061	**0.03**	-0.025	0.84	0.069	0.09
Meat	-0.040	0.77	-0.090	0.97	-0.014	0.66
Vegetable	0.031	0.31	-0.110	0.90	-0.003	0.47
Fruit	0.000	0.44	0.097	0.13	-0.066	0.78
Liquor	0.207	0.11	0.156	0.14	0.062	0.32
Cigarette	0.019	0.41	0.171	0.08	0.260	**0.04**

Using ANOISM to analyze if the similarity of flora composition in populations of groups who preferring or not preferring a food higher than which between groups.

Supplementary Table 3

id	Taxonomy	CD3	CD4	CD8	lgG	lgA	lgM	FTB	FDA
812a32cc2172f9044ebdb4c94170622f	D_4__Burkholderiaceae	0	0.49	0	0	0	0	Y	Y
95da764ae5d9fc9e31990661142b69f5	D_4__Enterobacteriaceae	0.45	0.62	0	0	0	0	Y	Y
264e6087ccd5bd1bddaf8155c8ea94fe	D_5__Escherichia-Shigella	0	0.44	0	0	0	0	Y	Y
f17d3da8eb98435912cf7cfb2d1bfbdb	D_5__Cutibacterium	0.48	0.49	0	0	0	0	Y	Y
ef817693535df14b3b9f5f946c64a5fd	D_6__alpha proteobacterium BIWA05	0	0.51	0	0	0	0	Y	Y
88d52f5c4d24256b438dc8c5b5e96b58	D_5__Ramlibacter	0	0.49	0	0	0	0	Y	Y
abf54b1694bf8bac8d6885505859a564	D_4__Enterobacteriaceae	0	0.45	0	0	0	0	Y	N
fb820cc6221c679ce57daa89b52650ce	D_6__uncultured bacterium	0	0.48	0	0	0	0	Y	Y
6af5f8ea781a98464482a500ff544d17	D_6__Bacteroides uniformis	0	0	0	0	0	0	Y	N
9c49d8c1d69daf5f9bfc7ff0cb592644	D_5__Streptococcus	0	0	0	0	-0.44	-0.68	Y	Y

List of ASVs which correlate with immune markers (|R|>0.3, P<0.05), total of 415 ASVs (part). FTB: familial transmissible bacteria, FDA: familial differential ASVs.
